# Protein Interactors and Trafficking Pathways That Regulate the Cannabinoid Type 1 Receptor (CB1R)

**DOI:** 10.3389/fnmol.2020.00108

**Published:** 2020-06-12

**Authors:** Alexandra Fletcher-Jones, Keri L. Hildick, Ashley J. Evans, Yasuko Nakamura, Jeremy M. Henley, Kevin A. Wilkinson

**Affiliations:** Centre for Synaptic Plasticity, School of Biochemistry, University of Bristol, Bristol, United Kingdom

**Keywords:** endocannabinoid system, cannabinoid type 1 receptor, trafficking, protein-protein interactions, synaptic regulation, retrograde synaptic signaling

## Abstract

The endocannabinoid system (ECS) acts as a negative feedback mechanism to suppress synaptic transmission and plays a major role in a diverse range of brain functions including, for example, the regulation of mood, energy balance, and learning and memory. The function and dysfunction of the ECS are strongly implicated in multiple psychiatric, neurological, and neurodegenerative diseases. Cannabinoid type 1 receptor (CB1R) is the most abundant G protein-coupled receptor (GPCR) expressed in the brain and, as for any synaptic receptor, CB1R needs to be in the right place at the right time to respond appropriately to changing synaptic circumstances. While CB1R is found intracellularly throughout neurons, its surface expression is highly polarized to the axonal membrane, consistent with its functional expression at presynaptic sites. Surprisingly, despite the importance of CB1R, the interacting proteins and molecular mechanisms that regulate the highly polarized distribution and function of CB1R remain relatively poorly understood. Here we set out what is currently known about the trafficking pathways and protein interactions that underpin the surface expression and axonal polarity of CB1R, and highlight key questions that still need to be addressed.

## Introduction

**FIGURE 1:**
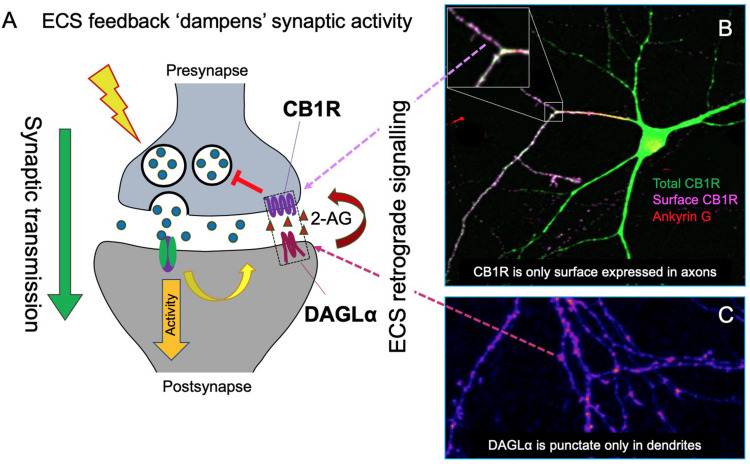
Schematic of the synaptic organization of the ECS. **(A)** The ECS is highly polarized and unlike nearly all neurotransmitter systems it acts retrogradely. The most abundant endocannabinoid, 2-arachidonoylglycerol (2-AG), is synthesized by the postsynaptic enzyme DAGLα and released to activate presynaptically localized cannabinoid receptors (CB1R) to suppress neurotransmitter release and reduce synaptic activity. **(B)** Representative image of total and surface expression of N-terminally GFP-tagged CB1R. CB1R is widely distributed in intracellular compartments in neurons, but only stably surface expressed in axons, particularly at the axon terminal. The image shows surface (non-permeabilized, purple) and total (permeabilized, green; colocalization shows as white) CB1R labeled with anti-GFP antibody. The axon was identified by Ankyrin G labeling, red (triple colocalization shows as yellow) (Fletcher-Jones et al. previously unpublished image). **(C)** Correspondingly, DAGLα is highly localized at the postsynapse. GFP-DAGLα (human) expressed in hippocampal neurons is highly punctate in dendrites consistent with postsynaptic localization (blue = low intensity, purple = high intensity) (Fletcher-Jones et al. previously unpublished image).

Information transfer at synapses is almost always mediated by neurotransmitter released from the presynaptic terminal activating specific postsynaptic receptors. The endocannabinoid system (ECS) is exceptional because it is a retrograde negative feedback system (Kreitzer and Regehr, 2001; Ohno-Shosaku et al., 2001; Wilson and Nicoll, 2001). The predominant endocannabinoid transmitter, 2-arachidonoylglycerol (2-AG), is synthesized in the postsynaptic membrane by diacylglycerol lipase-α (DAGLα). 2-AG then diffuses retrogradely across the synaptic cleft to stimulate cannabinoid type 1 receptors (CB1Rs) at the presynaptic membrane (Wilson and Nicoll, 2002; Castillo et al., 2012; Figure 1A). Activation of presynaptic CB1Rs suppresses neurotransmitter release to dampen down synaptic activity (Mackie and Hille, 1992; Yoshida et al., 2006; Chevaleyre et al., 2007; Jung et al., 2007; Uchigashima et al., 2007; Katona and Freund, 2008; Di Marzo, 2011). This unique architecture underpins the ECS function as a “sensor-feedback machine” that modulates synaptic activity.

The ECS plays key roles in multiple brain functions including synaptic plasticity the cellular basis of learning and memory (Castillo et al., 2012) the central regulation of food intake and energy expenditure (Cardinal et al., 2015) and attenuation of stress-induced glutamate release to counter excitotoxicity (Katona and Freund, 2008). Accordingly, ECS function and dysfunction is strongly implicated in a wide and diverse range of disorders including epilepsy, stroke, dementia, and obesity (Soltesz et al., 2015; Lu and Mackie, 2016; Busquets-Garcia et al., 2018).

## Cannabinoid Receptor 1 (CB1R)

Cannabinoid receptor 1 (CB1R) is a 473 amino acid G protein-coupled receptor (GPCR) highly expressed in the basal ganglia, hippocampus, cerebellum, and cortex (Herkenham et al., 1991). CB1R is a receptor for the endogenous cannabinoids (endocannabinoids) N-arachidonoylethanolamine (anandamide; AEA) and 2-arachidonylglycerol (2-AG) (Kendall and Yudowski, 2016) and mediates most effects of phytocannabinoids, the active components of cannabis, on the CNS.

Cannabinoid type 1 receptor surface expression in neurons is highly polarized to axons and presynaptic sites, where ligand binding results in the downregulation of presynaptic release through coupling to a variety of downstream signaling pathways (Figure 1). In cultured hippocampal neurons, CB1R has a wide intracellular distribution but its plasma membrane surface expression is highly polarized toward the axonal compartment (Figure 1; Irving et al., 2000; Coutts et al., 2001; Leterrier et al., 2006; McDonald et al., 2007a; Fletcher-Jones et al., 2019). CB1R displays a disto-proximal gradient of CB1R expression along the entire axonal plasma membrane (Simon et al., 2013; Wickert et al., 2018) with fine axons reported to exhibit highly punctate surface labeling, often co-localized with sites of synaptic contact (Irving et al., 2000; Coutts et al., 2001). Immunogold electron microscopy analysis of rat brain sections (Katona et al., 1999; Nyiri et al., 2005) and STORM super-resolution imaging (Dudok et al., 2015) detect CB1R predominantly at the presynaptic terminal, concentrated in the plasma membrane at axonal perisynaptic regions of both GABAergic and glutamatergic neurons (Katona et al., 1999, 2006; Kawamura et al., 2006). Notably, small, but functional, sub-populations of CB1R are also observed at the surface of soma and dendrites (Bacci et al., 2004; Marinelli et al., 2009) where they mediate slow self-inhibition, and at mitochondria, where they modulate bioenergetic processes (Benard et al., 2012; Koch et al., 2015). However, how differential targeting of distinct subpopulations to different cellular compartments occurs is unknown, and studies examining the mechanisms that establish and maintain the polarized surface expression of CB1R have provided evidence for contrasting models on how this occurs.

Here we briefly outline CB1R signaling, review the trafficking pathways proposed to mediate CB1R surface expression and axonal polarity, outline the role of the CB1R intracellular C-terminus in mediating these effects, and discuss the known protein interactors of CB1R that contribute to its trafficking and localization.

## CB1R Signaling

### Synaptic CB1R Signaling

**FIGURE 2:**
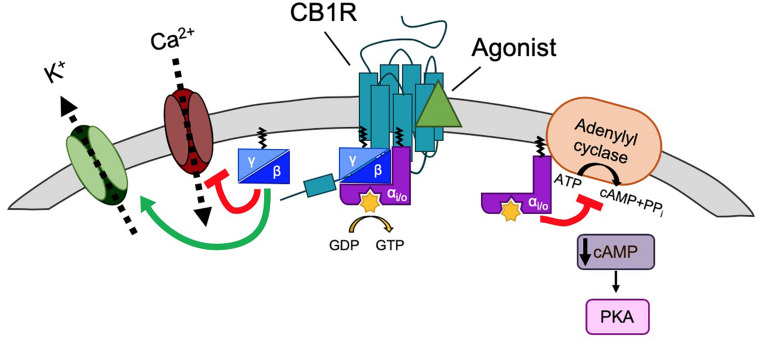
G-protein signaling of CB1R. Classically, CB1R signaling is mediated by G_i/o_ signaling. CB1R is activated by ligand (green triangle), generating a conformational change that allows for heterotrimeric G-protein (α, β, and γ subunits) binding. The receptor then acts as a guanine nucleotide exchange factor (GEF), activating Gα by GDP-to-GTP exchange. This triggers the dissociation of Gα from Gβγ. Gα_i/o_ inhibits adenylyl cyclase (AC), a membrane protein that catalyzes that conversion of ATP to cAMP+PP_i_, causing a decrease in cAMP levels and thus inhibiting the PKA phosphorylation pathway. Gβγ inhibits voltage gated Ca^2+^ channels (VGCCs) and activates G protein-coupled inwardly rectifying K^+^ channels (GIRKs).

Endocannabinoid system pharmacology and signaling is complex and is the subject of a number of recent reviews (Araque et al., 2017; Howlett and Abood, 2017; Hunter et al., 2017; Busquets-Garcia et al., 2018). Briefly, CB1R primarily activates G_i_ proteins, which cause downstream inhibition of cAMP accumulation (Howlett and Fleming, 1984; Howlett, 1987) mediated via the pertussis toxin-sensitive G_i_ α-subunit inhibition of adenylyl cyclase (Howlett et al., 1986; Howlett, 2004; Figure 2).

Downstream signaling of CB1R also modulates the activation of a number of kinases. Depletion of cytosolic cAMP levels leads to subsequent inactivation of the protein kinase A (PKA) phosphorylation pathway (Howlett, 2005) whereas the extracellular signal-regulated kinases (ERK), focal adhesion kinases (FAK), c-Jun N-terminal kinase 1/2, and the mitogen-activated protein kinases (MAPK) p42/p44 and p38 are all activated in response to CB1R stimulation (Turu and Hunyady, 2010).

As well as kinase signaling, the classical downstream effects of G_i_ coupled CB1R activation are increased K^+^ conductance, through activation of G protein-coupled inward rectifying K^+^ channels (K_ir_/GIRK), and decreased Ca^2+^ conductance, via inhibition of N- and P/Q-type voltage-gated calcium channels (Ca_V_), through interaction with the G_i_ βγ-subunit (Pan et al., 1996; Twitchell et al., 1997; Figure 2). In addition to G_i/o_ proteins, CB1R can also couple to G_s_, G_q_, and G_12/13_ depending on the cellular/protein context (Busquets-Garcia et al., 2018) for a review. Endocannabinoid activation of astroglial CB1R increases intracellular Ca^2+^ levels, likely via G_q_ protein coupling rather than G_i/o_ (Navarrete and Araque, 2008). Heterodimerisation of CB1R with dopamine D2 receptors can switch the signaling of CB1R and D2 from G_i/o_ to G_s_ when co-stimulated, generating the opposing effect: an increase in cAMP levels (Kearn et al., 2005). Different agonists can also elicit different patterns of G-protein coupling, e.g., WIN55212-2, but not Δ^9^-THC and ACEA, are able to stimulate G_12/13_ in mouse cortex (Diez-Alarcia et al., 2016).

Endocannabinoid system signaling downstream of CB1R is crucial for both short- and long-term forms of synaptic plasticity. The CB1R-mediated decrease in presynaptic intracellular Ca^2+^ results in a short-term suppression of inhibition (depolarization-induced suppression of inhibition; DSI) or excitation (DSE), at GABAergic (Wilson and Nicoll, 2001; Ohno-Shosaku et al., 2002) and glutamatergic (Kawamura et al., 2006; Ohno-Shosaku et al., 2007) synapses, respectively, and ECS signaling plays well-established roles in certain forms of long-term depression (LTD) and long-term potentiation (LTP) (Wilson and Nicoll, 2001; Marsicano et al., 2002; Robbe et al., 2002; Chevaleyre and Castillo, 2004; Chevaleyre et al., 2007; Heifets and Castillo, 2009; Puighermanal et al., 2009; Han et al., 2012). Although primarily studied in the hippocampus and cerebellum, ECS signaling has also been implicated in the regulation of cortical, striatal, and limbic circuits (Di Marzo, 2011; Katona and Freund, 2012; Luchicchi and Pistis, 2012).

### Signaling From Intracellular CB1R

This review focuses on the trafficking and polarized surface expression of CB1R but, intriguingly, CB1R expressed at the plasma membrane comprises only ∼20% of total CB1R (McIntosh et al., 1998; Leterrier et al., 2006; Rozenfeld and Devi, 2008). Intracellular CB1R is particularly enriched within the endosomal system (McIntosh et al., 1998; Leterrier et al., 2006; Rozenfeld and Devi, 2008). Indeed, since CB1R ligands are lipophilic, and thus membrane permeable, it has been proposed that these endosomal CB1Rs are functional since they associate with Gα_i_ in Rab7-positive late-endosomal fractions (Rozenfeld and Devi, 2008) and the agonist WIN55,212-2 can still induce ERK1/2 phosphorylation even when surface CB1Rs are blocked by the membrane-impermeable antagonist hemopressin (Rozenfeld and Devi, 2008). Consistent with this possibility, intracellular injection of the endocannabinoid anandamide into HEK293 cells expressing CB1R induced Ca^2+^ release from intracellular stores (Brailoiu et al., 2011). Furthermore, activation of CB1Rs reported to associate with the mitochondrial outer membrane reduce mitochondrial respiration (Benard et al., 2012; Koch et al., 2015) and are implicated in for certain types of memory (Hebert-Chatelain et al., 2016). For recent reviews see Djeungoue-Petga and Hebert-Chatelain (2017), Busquets-Garcia et al. (2018).

## CB1R Traffic Through the Secretory Pathway

### ER Membrane Insertion

**FIGURE 3:**
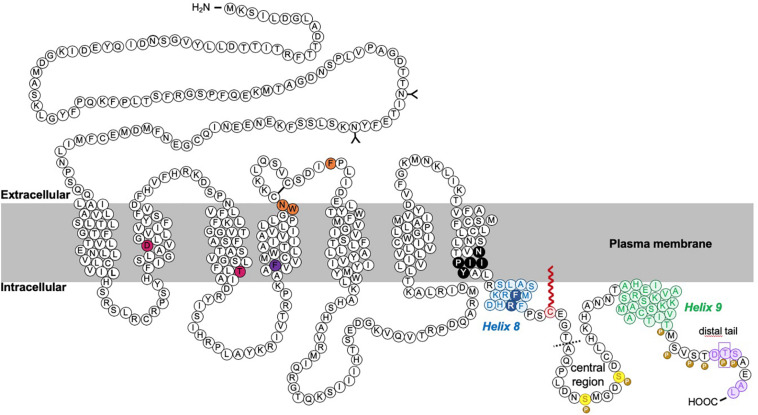
Schematic of rat CB1R. The predicted topology of rat CB1R based on hydrophobicity plots and analysis from crystal structures of human CB1R (97% identity with rat CB1R) and rhodopsin-family GPCRs. Sites of post-translational modification via N-terminal glycosylation (Song and Howlett, 1995) or palmitoylation (Oddi et al., 2018, 2017, 2012) are indicated by the (Y) symbol and a red zigzag line, respectively. Two residues located within the 2nd and 3rd transmembrane domains (TM2/TM3), required for constitutive (Jin et al., 1999) internalization are shown in pink (Roche et al., 1999; D’Antona et al., 2006). Orange residues denote putative amino acids involved in ER exit and surface expression (Ahn et al., 2009a). The F238 residue highlighted in dark purple modulates association with lipid rafts. The black residues indicate the sites of the highly conserved “elbow” shaped NPXXY motif. Within the C-terminal tail, two helical structural motifs, helix 8 (*H8*) and helix 9 (*H9*) are highlighted in blue and green, respectively (Ahn et al., 2009b; Stadel et al., 2011). Palmitoylation of C416 (red zigzag line) aids Helix 8 association with the inner leaflet of the plasma membrane. Phosphorylation of two serine residues (yellow) in the central region of the intracellular C-terminus mediate desensitization (Hsieh et al., 1999; Daigle et al., 2008b; Straiker et al., 2012b). However, further phosphorylation of 6 serine/threonine residues in the distal tail (each indicated by the “P” symbol) is required for internalization. GASP1 likely binds the conserved F409/R410 motif in *H8*, although the distal tail has also been reported to bind GASP1. The motifs required for CRIP1a binding are shown in purple. Phosphorylation of T468 (purple rectangle) decreases affinity for CRIP1a, allowing β-arrestin-2 binding. The exact binding site of SGIP1 is unknown, but it binds downstream of A420 (dotted line). A summary of mutations is provided in Table 1.

Cannabinoid type 1 receptor is trafficked to the plasma membrane via the secretory pathway but, like many other GPCRs, CB1R does not have a canonical, cleavable signal peptide (Andersson et al., 2003). Rather, the first transmembrane domain is thought to act as a “reverse anchoring sequence” which is recognized by the signal recognition particle (SRP) Sec61 pathway in the ER membrane (Andersson et al., 2003; Nordstrom and Andersson, 2006). The N-terminal domain is then translocated across the ER membrane in a C-N terminal manner (Rutz et al., 2015) where it is modified by N-linked glycosylation at two sites (N78, N84) [(Song and Howlett, 1995; Nordstrom and Andersson, 2006); unless otherwise stated, all residue numbers used throughout refer to the rat CB1R sequence; Figure 3]. The unusually large size of N-terminal tail of CB1R (117 amino acids) impedes its reverse translocation across the membrane, raising the possibility that a substantial proportion of nascent CB1R could be misfolded and degraded by the proteosome (Andersson et al., 2003; Nordstrom and Andersson, 2006). Indeed, truncation of the large N-terminus, or inclusion of a signal peptide, can greatly enhance receptor stability and cell surface targeting with no effect on agonist binding or signaling [(Andersson et al., 2003; McDonald et al., 2007a) a list of published mutants of CB1R and a brief overview of their effects on trafficking, surface expression and signaling are shown in Table 1]. Endogenous truncation of the N-terminal tail has also been reported, likely occurring during the short amount of time that the N-terminus is exposed to the cytoplasm before translocation (Nordstrom and Andersson, 2006). Interestingly, the first 22 residues of the N-terminus have been reported to constitute a mitochondria targeting sequence (Hebert-Chatelain et al., 2016), suggesting the N-terminus of CB1R represents an important determinant of receptor stability and targeting.

**TABLE 1:** Summary of published CB1R mutants.

	Amino Acids	cDNA	Tag	Cell Type	Surface Expression	Trafficking Properties?	Signaling properties?	References
*Ct-tail truncation*	1–400	human	–	(SCG) neurons and HEK293 cells	Unaltered (in HEK293)	–	Abolished G-protein coupling	Nie and Lewis, 2001b
	1–417	human	–	(SCG) neurons and HEK293 cells	Unaltered (in HEK293)	Enhanced constitutive internalization	↓ G-protein activation	Nie and Lewis, 2001a, b
	↑14	rat	–	AtT20	Unaltered	Prevented agonist induced internalization	–	Hsieh et al., 1999
	↑14	rat	–	HEK293	Unaltered	*No effect* on agonist induced internalization	–	Hsieh et al., 1999
	↑14	rat	Nt-GFP	rat hippocampal neurons	Traffics to surface (axonal polarized)	–	–	McDonald et al., 2007a, b
	↑13	rat	–	mouse hippocampal neurons (CB1R knockout)	–	–	No desensitization	Straiker et al., 2012b
*Ct tail deletion*	Δ418–439	rat	–	Xenopus oocytes	–	Prevented β-arrestin-2/GRK mediated down-regulation	No desensitization	Jin et al., 1999
	Δ419–460	rat	–	mouse hippocampal neurons (CB1R-/-)	–	–	No effect on desensitization	Straiker et al., 2012b
	ΔH9 (441–461)	rat	Nt-SBP-GFP	rat hippocampal neurons	Reduced in axons and dendrites	↑ delivery to dendrites ↑ agonist-induced endocytosis	↓ERK1/2 phosphorylation	Fletcher-Jones et al., 2019
*Ct tail point mutation*	L404F in H8	human	–	HEK293	Unaltered	↑rate of internalisation	↓ G-protein activation, ↓Ca^2+^ inhibition	Anavi-Goffer et al., 2007
	φ-A triple mutant in H8 (L404A/F408A/F412A)	human	Ct-GFP	HEK293	ER retention (localisation perturbed)	–	↓ ligand binding and ↓ G-protein activation	Ahn et al., 2010
	S426A/S430A (phospho-null)	rat	–	Xenopus oocytes and AtT20 cells	Unaltered	Prevent β-arrestin-2/GRK mediated down-regulation	No desensitization	Jin et al., 1999
	mutation of last 6 S/T-A (phospho-null)	rat	–	HEK293	Unaltered	Attenuates internalisation	–	Daigle et al., 2008b
	mutation of last 6 S/T-A (phospho-null)	rat	–	mouse hippocampal neurons (CB-/-)	–	–	No desensitization	Straiker et al., 2012b
	C415	human	Ct-GFP	HEK293 and 5HSY-5Y	Reduced surface localisation	↑ diffusional mobility	↓G protein coupling/activation (Gαi/o)	Oddi et al., 2012
	D467A;T468A; S469A;A472G; L473A	rat	–	HEK293		No CRIP1a-dependent reduction in internalization	–	Mascia et al., 2017
*Nt*	Nt truncations (↑64,↑80,↑89,ssNt)	human	Nt-myc	HEK293	↑ surface localisation	Facilitates ER exit	–	Andersson et al., 2003
*TM domains*	2nd TM domain (D164N)	rat	–	AtT20	Unaltered	No internalization	KIR current potential inhibited	Roche et al., 1999
	2nd TM domain (D164N)	rat	–	(SCG) neurons	Unaltered	No constitutive internalization	–	Nie and Lewis, 2001b
	2nd TM domain (D164N)	rat	Nt-GFP	rat hippocampal neurons	Traffics to surface (axonal polarized)	–	–	McDonald et al., 2007b
	3rd TM domain (T210A/I)	rat	Ct-GFP	HEK293	↑/↓surface localisation (T210A/I) ↓surface localisation (T210I)	↓ constitutive internalization (T210A) ↑ constitutive internalization (T210I)	T210I: hyperactive T210A: hypoactive	D’Antona et al., 2006
	3rd TM domain (T210A/I)	rat	Nt-FLAG, Ct-GFP	rat hippocampal neurons	Non-polarized (T210A) ↓surface localisation (T210I)	↑ constitutive internalization (T210I)	T210I: hyperactive T210A: hypoactive	Simon et al., 2013
	4th TM domain F238L	rat	Nt-HA	HEK293; Rat hippocampal neurons	↑lipid raft association ↓surface localisation ↑axonal polarity	↑ lipid raft-mediated constitutive internalization	–	Wickert et al., 2018
*EC loop*	2nd EC Loop (TM4-TM5)	human	Ct-GFP	HEK293	↓ surface expression	ER retention (W255A, N256A, F268A)	ligand binding perturbed	Ahn et al., 2009b

### TGN Sorting

Selective sorting of proteins to the correct subcellular domain is crucial but, conceptually, establishing axonal polarity is more complex than establishing dendritic polarity since both dendritic and axonal cargos are synthesized in the somatodendritic compartment [although see (Gonzalez et al., 2018; Luarte et al., 2018) regarding the possibility of an axonal local secretory pathway]. The TGN represents an important “hub” in determining polarized protein trafficking, with the target domain of the TGN-derived vesicles acting as a major determinant of the route taken by the cargo to the axonal surface. While several sorting signals and adaptors have been described for dendritic cargo, the mechanisms underpinning polarized sorting of proteins to axons are less well defined (Lasiecka and Winckler, 2011; Bentley and Banker, 2016).

#### Evidence for Non-polarized Delivery of CB1R From the TGN

**FIGURE 4:**
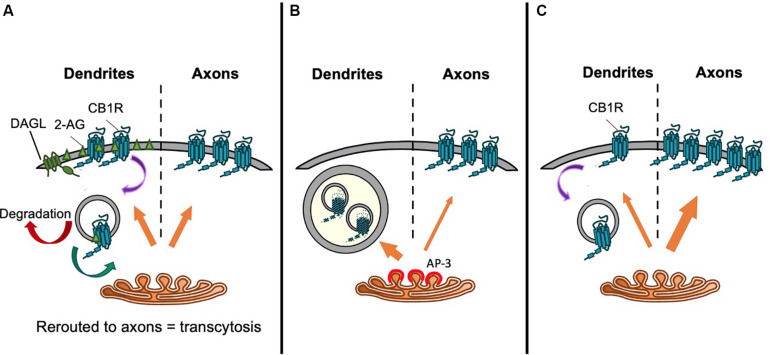
Proposed trafficking pathways that establish CB1R axonal polarization. Schematics representing **(A)** Model 1: CB1R is delivered to the dendritic and axonal surface indiscriminately. The presence of DAGLα exclusively on the dendritic surface causes a polarized production of 2-AG with high levels in the dendritic membrane. Therefore, *de novo* CB1R delivered to the dendritic membrane is immediately activated and internalized. Internalized CB1R is either degraded or rerouted to distal end of axons, a process referred to as transcytosis. On the other hand, the absence of DAGLα in the axonal compartment means that newly delivered CB1R is retained on the axonal surface. This results in the axonally polarized distribution, where it is available to be bound by retrogradely crossing endocannabinoids produced under specific synaptic conditions (Leterrier et al., 2006). **(B)** Model 2: Constitutive sorting to somatodendritic lysosomes. CB1R is constitutively sorted by AP-3 from the TGN to somatodendritic lysosomes. By an unknown mechanism, CB1R can be rescued from degradation and rerouted to axons. It is possible that this mechanism is also AP-3-dependent, since in AP-3 sorts cargo from the TGN to axons in *C. elegans* (Rozenfeld and Devi, 2008; Li et al., 2016). **(C)** Model 3. Secretory pathway bias. CB1R is preferentially targeted via the secretory pathway to the axonal membrane. Polarity is enhanced by immediate removal of *de novo* CB1R from dendritic surface (Fletcher-Jones et al., 2019).

Brefeldin A (BFA) reversibly prevents secretory pathway trafficking by disrupting the Golgi (Hunziker et al., 1991; Graham et al., 1993). Using a BFA block and release protocol in cultured neurons to monitor CB1R forward traffic, it has been reported that CB1R is non-discriminately delivered to both the somatodendritic and axonal membrane (Leterrier et al., 2006). Following BFA washout to allow synchronous release of receptors from the Golgi, within 4–8 h more than 50% of neurons exhibited surface expressed CB1R either uniformly throughout the neuron, or on the somatodendritic membrane, suggesting that receptors are sorted from the TGN in a non-polarized manner (Leterrier et al., 2006). 24 h after BFA washout, two-thirds of neurons displayed the expected axonally polarized surface expression. These data were interpreted to indicate that differential rates of constitutive endocytosis in axons versus dendrites lead to the establishment of axonal polarity (see “Differential Internalization of CB1R” section below), rather than polarized trafficking through the secretory pathway (Leterrier et al., 2006; Figure 4A).

#### Targeting to Late Endosomes/Lysosomes From the TGN

An alternative suggestion is that CB1R does not reach the somatodendritic surface after egress from the TGN. Rather, CB1R is constitutively targeted primarily to late endosomes/lysosomes (Figure 4B). In the N18TG2 neuroblastoma cell line, newly synthesized CB1R is rapidly degraded without reaching the membrane, with most intracellular CB1R having a half-life of less than 5 h (McIntosh et al., 1998). Moreover, in primary neurons, a significant proportion of endogenous CB1R colocalises with late endosomal/lysosomal markers (Rozenfeld and Devi, 2008). Interestingly, the delta subunit of AP-3, an adaptor complex that mediates trafficking between the TGN and lysosomes (Odorizzi et al., 1998; Scales and Scheller, 1999; Guardia et al., 2018) immunoprecipitates with CB1R, suggesting that CB1R associates with AP-3 (Rozenfeld and Devi, 2008). Indeed, AP-3δ knockdown increased the surface expression of CB1R in both Neuro2A cells and in primary hippocampal neurons (Rozenfeld and Devi, 2008) suggesting that interaction with AP-3δ acts to localize CB1R to late endosomes/lysosomes and restrict somatodendritic surface expression.

AP-3 isoforms have been proposed to play a number of specialized roles in neurons including biogenesis of synaptic vesicles and sorting of transmembrane proteins from endosomes to synaptic vesicles (Nakatsu et al., 2004; Salazar et al., 2004). Moreover, AP-3 may mediate axonal cargo secretory vesicle budding from the TGN, analogous to the better understood role of AP-1 and AP-4 in budding of dendritically destined secretory vesicles (Danglot and Galli, 2007; Li et al., 2016; Guardia et al., 2018), raising the, as yet unconfirmed, possibility that AP-3 may play a role in directing axonal delivery of CB1R, in addition to its role in mediating late endosomal/lysosomal targeting.

#### Direct Preferential Trafficking of CB1R to the Axon

While these proposed non-polarized and AP-3δ-based targeting mechanisms are of undoubted interest, they are limited by the fact that they are based on experiments that block the transit of multiple proteins through the secretory pathway. BFA blocks any cargo from trafficking and, furthermore, has been shown to also affect endocytosis and recycling (Miller et al., 1992; Wood and Brown, 1992), and AP-3δ knockdown affects any cargo sorted by AP-3.

Most recently, a targeted, retention using selective hooks (RUSH) approach indicated that CB1R is preferentially delivered to the axonal membrane by the secretory pathway (Fletcher-Jones et al., 2019; Figure 4C). CB1R was engineered to incorporate an N-terminal fluorescent protein and streptavidin binding peptide (SBP) tag which was anchored in the ER by the expression of an ER-localized streptavidin-KDEL “hook” (Boncompain et al., 2012; Evans et al., 2017). The addition of biotin, which has a greater affinity for streptavidin than SBP (Keefe et al., 2001), outcompetes the hook-reporter interaction and the reporter is synchronously released and trafficked by bulk flow through the secretory system to the plasma membrane. The progress of the reporter can be monitored by live imaging of the fluorescent protein or by fixed immunostaining for SBP and/or the fluorescent protein (Evans et al., 2017). These experiments revealed that after release from the ER, intracellular CB1R is detected in the axon as soon as 25 min after release, and significantly more *de novo* CB1R is delivered by the secretory pathway to the axonal membrane than the somatodendritic membrane (Fletcher-Jones et al., 2019). These data indicate that CB1R polarity is established early in the secretory pathway by directed trafficking of CB1R-containing vesicles to axons.

## CB1R and the Endosomal System

While polarity can be established by directed trafficking from the TGN, receptor polarity can be maintained by differential rates of endocytosis in different neuronal compartments. CB1R undergoes both agonist-dependent and constitutive endocytosis, and differences in the rate of endocytosis between axons and dendrites have been proposed to maintain the axonally polarized surface expression of CB1R.

### Mechanisms of Agonist-Induced Endocytosis

Like other GPCRs, internalization of CB1R is dynamically regulated by receptor stimulation. Agonist profiling studies of CB1R in cell lines indicate that potent agonists (e.g., WIN55,212-2, CP55,940 and HU 210) cause more rapid internalization than Δ^9^-THC, the psychoactive component of cannabis, or endogenous cannabinoid analogs, such as methanandamide (Hsieh et al., 1999). It is important to note, however, that the pattern and rate of endocytosis varies according to cell type and, in particular, endocytosis proceeds more slowly in neurons than in clonal cell lines (Coutts et al., 2001; Leterrier et al., 2006). This likely reflects differences in the molecular mechanisms which direct receptor trafficking according to the cell type or membrane subdomain. For example, truncation of last 14 amino acids of CB1R markedly reduces activity-dependent internalization in AtT20 cells but does not affect rates of internalization in HEK293 cells (Hsieh et al., 1999; Daigle et al., 2008a), suggesting cellular context represents an important determinant of CB1R endocytosis. Indeed, given that difference in the rate of constitutive endocytosis between different neuronal compartments has been proposed to contribute to the polarized expression of CB1R, it appears that context, even within the same cell, plays an important role in CB1R behavior.

**FIGURE 5:**
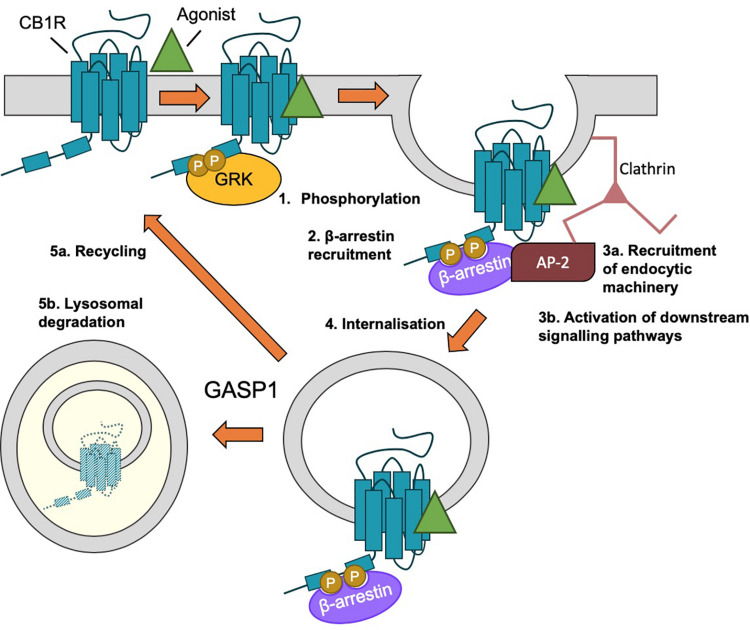
β-arrestin-mediated internalization of CB1R. (1) Following agonist binding, GPCR kinases (GRKs) phosphorylate the C-terminal tail of CB1R. (2) Phosphorylation of ctCB1R leads to recruitment of β-arrestins. (3) β-arrestin recruitment (a) acts as scaffold for recruitment of AP-2 and clathrin and (b) elicits the activation of downstream kinase cascades, most classically, the activation of ERK1/2. (4) The desensitized CB1R is internalized by clathrin-mediated endocytosis (CME). (5) Endosomal CB1R is either recycled (a) or sorted by GASP1 binding for lysosomal degradation (b), depending on the concentration and duration of agonist.

Agonist-induced internalization of GPCRs, including of CB1R, generally occurs as a result of receptor phosphorylation and β-arrestin binding (see “β-Arrestin” section below), followed by clathrin-mediated endocytosis (CME) (Ahn et al., 2013b; Blume et al., 2016; Figure 5). However, blocking CME only partially impairs CB1R internalization (Keren and Sarne, 2003; Wu et al., 2008). An alternative and/or parallel mechanism is caveolin-mediated endocytosis of proteins localized to lipid rafts (Razani et al., 2002). Lipid rafts are specialized microdomains of plasma membrane rich in sphingolipids and cholesterol (Allen et al., 2007) and plasma membrane expressed CB1R has been reported to reside in lipid rafts (Sarnataro et al., 2005; Asimaki and Mangoura, 2011). Indeed, membrane cholesterol depletion using methyl-β-cyclodextrin (MβCD), which disrupts lipid rafts, is an effective way of preventing CB1R endocytosis (Keren and Sarne, 2003; Leterrier et al., 2004, 2006) and a mutation that increases lipid raft association of CB1R (CB1R-F238L) increases constitutive endocytosis of CB1R (Wickert et al., 2018). Association with lipid raft domains has also been proposed to influence receptor signaling and ligand binding (Keren and Sarne, 2003; Bari et al., 2005, 2008; Wu et al., 2008) and this association has been reported to rely on palmitoylation of cysteine residue C416 in rat CB1R (see “Palmitoylation of ctCB1R” section below).

### Differential Internalization of CB1R – A Mechanism for Maintaining Polarity?

Cannabinoid type 1 receptor displays high levels of constitutive activity due to significant endocannabinoid tone in brain and other tissues (Nie and Lewis, 2001a; Pertwee, 2005; Howlett et al., 2011) that is sensitive to the application of antagonists such as SR 141716A (Bouaboula et al., 1997; Pan et al., 1998) or AM251/AM281 (Leterrier et al., 2004, 2006). Consequently, CB1R undergoes constitutive internalization, and this has been proposed as a mechanism for the polarized surface distribution of CB1R in neurons, since it has been reported that rapid rates of constitutive endocytosis in somatodendritic compartments leads to an accumulation of CB1R at the axonal plasma membrane (Leterrier et al., 2006; McDonald et al., 2007a; Simon et al., 2013). More specifically, monitoring internalization of CB1R in cultured hippocampal neurons by live incubation with an antibody recognizing an extracellular epitope (antibody feeding), followed by stripping remaining surface antibody with a low pH wash, demonstrated an enhanced rate of receptor endocytosis in the somatodendritic compartment compared to axons (Leterrier et al., 2006). Furthermore, blockade of receptor endocytosis by overexpression of a dominant-negative dynamin 1 disrupts CB1R polarity and increases its somatodendritic surface expression (Leterrier et al., 2006; Bohn, 2007), suggesting these differential rates of endocytosis are capable of supporting polarized surface expression.

Whether this enhanced dendritic internalization is dependent on receptor activity, or on the conformational state of the receptor, has produced conflicting results. It has been reported that preventing the receptor adopting an active conformation using the inverse agonist AM281 leads to an increase in somatodendritic surface expression of CB1R and therefore a loss of axonal polarity (Leterrier et al., 2006). Furthermore, “locking” the receptor in a hypoactive conformation by incorporation of a T211A mutation (D’Antona et al., 2006) resulted in a loss of axonal polarity (Simon et al., 2013). Indeed, somatodendritic CB1R exhibits a high level of constitutive activity as a result of locally produced 2-AG (Ladarre et al., 2014) and reducing constitutive activity in response to endogenously produced 2-AG using the DAGLα inhibitor THL results in an upregulation of CB1R on the somatodendritic surface (Turu et al., 2007). Together, these studies support a role for enhanced endocytosis from the somatodendritic compartment occurring in response to high levels of constitutive activity of the receptor (Figure 4A). In contrast, other studies detected no effect of inverse agonists on CB1R axonal polarity (Coutts et al., 2001; McDonald et al., 2007a), and reported that CB1R mutants that display defective constitutive activity [CB1R-D164N; (Nie and Lewis, 2001a)] or that lack agonist-induced endocytosis in heterologous cells [a mutant lacking the last 14 amino acids (Hsieh et al., 1999)] exhibit normal axonal polarity (McDonald et al., 2007a).

Using antibody feeding to monitor surface expression during the RUSH forward trafficking assay, Fletcher-Jones et al. (2019) found that newly delivered axonal CB1R exhibited relatively stable surface expression, whereas what little CB1R was delivered to the dendritic plasma membrane was rapidly removed by endocytosis (Fletcher-Jones et al., 2019; Figure 4C). Notably, however, there was only a small, non-significant trend toward increased dendritic surface expression following treatment with the inverse agonist AM281. This study therefore supports a model whereby the main driving force for axonal polarity is preferential delivery to the axonal plasma membrane, whereas endocytosis, constitutive or agonist-induced, acts to maintain and enhance polarity.

Thus, an emerging consensus in the field is that differential rates of endocytosis between axons and dendrites play an important role in maintaining CB1R polarity. Furthermore, there is accumulating evidence for a role of constitutive receptor activity in this process, although the findings are conflicting, potentially due to the different experimental systems (for example, the age and culturing methods of primary neurons) and CB1R constructs used (for example the use of N- versus C-terminally tagged receptors). Clearly, further work will be required to reconcile these differences. Nonetheless, how the different rates of endocytosis between neuronal compartments occurs mechanistically is an important outstanding question in the field. It remains possible that compartment-specific interactions that stabilize receptor surface expression, or enhance endocytosis, in distinct compartments may underlie the differential endocytosis of CB1R in axons versus dendrites.

## Post-Endocytic Sorting of CB1R

### Degradation

After activation, most GPCRs are endocytosed from the cell surface into low pH endosomes that facilitate ligand detachment, before the receptor is recycled back to the surface or trafficked for degradation (Ferguson, 2001; Figure 5). Several studies have examined CB1R recycling following agonist-induced internalization, using immunocytochemistry and live antibody feeding protocols, to selectively label surface expressed exogenous CB1R in cell lines to follow their endocytic fate (Hsieh et al., 1999; Sim-Selley et al., 2006; Martini et al., 2007, 2010; Tappe-Theodor et al., 2007; Grimsey et al., 2010; Blume et al., 2016). Together, these studies indicate that relatively short agonist exposure (<20 min) and/or low agonist concentrations favor recycling. Increasing the concentration or duration of agonist application promotes receptor degradation, resulting in recovery by newly synthesized receptors, reduced surface levels, and accumulation within lysosomal compartments. Indeed, prolonged agonist exposure and the subsequent down-regulation and desensitization of CB1R, in combination with changes in receptor G protein coupling and endocannabinoid content, has been implicated in the tolerance and dependence associated with prolonged cannabis use (Mato et al., 2005; Martini et al., 2010).

### Transcytosis

What happens to CB1R following internalization from the somatodendritic membrane is unclear. However, the Lenkei group have presented evidence that CB1R may traffic in a transcytotic manner, with endosomal somatodendritic CB1R being sorted and sent to the axonal membrane, particularly to more distal areas (Simon et al., 2013). To investigate this, they grew primary neurons in microfluidic chambers, which allow for the separation of the somatodendritic and axonal domains. Interestingly, a small amount of N-terminal antibody fed into the somatodendritic compartment of the chamber was detected after 4 h on the axonal membrane, especially in the very distal end of the axon (Simon et al., 2013) suggesting that receptors that briefly reach the somatodendritic surface are subsequently rerouted to the axonal membrane. However, the pathways that mediate this transcytotic sorting have yet to be established.

## Roles of the CB1R C-Terminal Domain

In common with many other neurotransmitter receptors, multiple protein interactions and post-translational modifications occur at the intracellular C-terminal region of CB1R (ctCB1R; Figure 3). NMR spectroscopy and circular dichroism analysis has revealed that ctCB1R contains two α-helical domains, known as Helix 8 (*H8)* and Helix 9 (*H9*), respectively (Ahn et al., 2009b). These helices are amphipathic and likely associate with the plasma membrane along their non-polar faces (Ahn et al., 2009b; Stadel et al., 2011).

A recent report highlighted the importance of the CB1R C-terminal domain in polarized trafficking and surface expression in cultured neurons (Fletcher-Jones et al., 2019). The single-pass membrane protein CD4 has no intrinsic localization signals and is normally surface expressed in a non-polarized manner (Garrido et al., 2001; Fache et al., 2004). However, a CD4-ctCB1R chimera displays a markedly more axonally polarized surface distribution than CD4 alone. Furthermore, CD4-ctCB1R was internalized more compared to CD4 in dendrites but not in axons, indicating ctCB1R plays a role in establishing the differential endocytosis described previously (Fletcher-Jones et al., 2019). Importantly, however, axonal surface expression of CD4-ctCB1R was not completely polarized, suggesting that other localization motifs or signals potentially induced by agonist binding are required for full polarization.

### Helix 8 *(H8)*

Helix 8 is a conserved feature of class A GPCR C-termini (Han et al., 2001) and has been identified in crystal structures across a range of GPCR subtypes. NMR studies demonstrate that, in a variety of GPCRs, *H8* forms an amphipathic helix that associates with the plasma membrane via its hydrophobic face, and may also contact intracellular loops in an activity-dependent manner (Murakami and Kouyama, 2008; Ahn et al., 2010; Stadel et al., 2011). It is positioned immediately downstream of another highly conserved motif, the membrane imbedded NPXXY motif located at the end of TM7. In rhodopsin, the NPXXY motif forms a bending “elbow region,” allowing *H8* to contact the plasma membrane, providing structural constraints on *H8*, and mediating structural rearrangements during receptor conformational switches from active to inactive states (Fritze et al., 2003).

In various GPCRs, *H8* has been implicated in receptor homodimerization (Salahpour et al., 2004; Knepp et al., 2012; Parmar et al., 2017), ER exit (Salahpour et al., 2004; Parmar et al., 2017), surface expression (Feierler et al., 2011; Spomer et al., 2014; Pandey et al., 2019), as a site of G protein coupling (Delos Santos et al., 2006; Kaye et al., 2011; Kuramasu et al., 2011; Kawasaki et al., 2015; Markx et al., 2019), and in β-arrestin binding (Feierler et al., 2011; Kirchberg et al., 2011; Kang et al., 2015; Yang et al., 2019) and subsequent internalization (Faussner et al., 2005; Aratake et al., 2012). Specifically in CB1R, disruption of *H8* helicity and hydrophobicity impairs CB1R trafficking, causing it to accumulate in the ER (Ahn et al., 2010). However, the other roles *H8* plays in the trafficking, signaling and protein interaction profile of CB1R are currently unknown.

### Helix 9 (*H9*)

Until recently, the functional significance of *H9* has been enigmatic, with only two other GPCRs, squid rhodopsin (Murakami and Kouyama, 2008) and the bradykinin receptor (Piserchio et al., 2005) reported to have an analogous structural domain (Stadel et al., 2011). However, studies of squid rhodopsin suggest an association of *H9* with the plasma membrane via interactions with other cytoplasmic regions including *H8* (Shimamura et al., 2008). For CB1R, evidence from NMR spectroscopy suggests that *H9*, like *H8*, lies along the inner-membrane surface, associating with the lipid bilayer via a cluster of hydrophobic residues on the non-polar face of the helix (Ahn et al., 2009b, 2010). As suggested for squid rhodopsin (Murakami and Kouyama, 2008) and the bradykinin receptor (Piserchio et al., 2005) the polar face of *H9* may further serve as a Gα_q_-binding site.

Most recently, evidence has shown that *H9* plays multifaceted roles in the polarized delivery and membrane retention of CB1R, and in the downstream activation of ERK1/2 following stimulation with the CB1R agonist arachidonyl-2′-chloroethylamide (ACEA) (Fletcher-Jones et al., 2019). A CB1R mutant lacking *H9* (CB1R^ΔH9^) displayed increased secretory pathway delivery to dendrites, indicating that *H9* contributes to polarized delivery by restricting sorting to dendrites. Furthermore, CB1R^ΔH9^ was less surface expressed and endocytosed more, in both axons and dendrites, than CB1R^WT^. Application of the inverse agonist AM281 reversed the increased internalization, suggesting that *H9* stabilizes CB1R against agonist-induced internalization. Furthermore, deletion of *H9* also reduced ERK1/2 activation in response to ACEA, suggesting *H9* is required for the full signaling capacity of the receptor (Fletcher-Jones et al., 2019). However, future work is needed to define the structural contribution of *H9*, or *H9* interacting proteins, that mediate these effects.

### Palmitoylation of ctCB1R

The presence of a palmitoylation site immediately downstream of *H8* (C416 in rat CB1R) is conserved amongst class A GPCRs (Sensoy and Weinstein, 2015) and computational modeling of CB1R, as well as the dopamine D2 receptor and the β2-adrenergic receptor, suggests that palmitoylation of this conserved residue enhances the stability and membrane/lipid raft association of the amphipathic *H8* (Oddi et al., 2017). Mutation of the corresponding cysteine in human CB1R to an alanine residue (C415A) prevented palmitoylation of CB1R in HEK293 cells (Oddi et al., 2012). Moreover, analysis of the palmitoylation state of CB1R isolated from plasma membrane-enriched P2 membrane fractions derived from rat forebrain indicated that the majority of plasma membrane CB1R is palmitoylated (Oddi et al., 2012). When this residue is de-palmitoylated, membrane association of *H8* ceases to be energetically favorable, so the helix unravels, causing some interaction sites to be lost and exposing other interaction sites to the aqueous domain (Sensoy and Weinstein, 2015). Overall, characterization of the non-palmitoylatable CB1R mutant suggests that palmitoylation at this site affects CB1R trafficking, localization, and signaling (Oddi et al., 2012, 2017, 2018). Specifically, blocking palmitoylation reduced plasma membrane expression, increased diffusional mobility, and prevented agonist-induced internalization of the receptor in SH-SY5Y and HEK293 cells, effects attributed to decreased association of CB1R with lipid rafts and caveolin-1 (Oddi et al., 2012, 2017). Furthermore, CB1R signaling was also diminished in the C416A CB1R mutant due to the instability of *H8*, which prevented the interaction of CB1R with G proteins and β-arrestins (Oddi et al., 2012, 2017, 2018).

### Proteins That Interact With ctCB1R

Although not as extensively characterized as many other neurotransmitter receptors, there have been a number of studies investigating the proteins that interact with the C-terminal domain of CB1R. The best characterized of the currently identified interacting proteins are discussed below. Of these, β-arrestins and GASP1 are relatively generic in that they play important roles in pathways common to the regulation of multiple GPCRs. CRIP1 and SGIP1, however, are more selective for CB1R, suggesting that these could be suitable candidates for manipulation to specifically intervene in CB1R localization and function. Importantly, however, the proteins already identified are certainly not a comprehensive list and future studies to identify and validate novel protein-protein interactions will provide important information about the molecular mechanisms of CB1R trafficking and polarity.

#### β-Arrestins

β-arrestins 1 and 2 regulate agonist-induced internalization and desensitization of multiple GPCRs (Moore et al., 2007) and CB1Rs have been reported to undergo β-arrestin-mediated agonist-dependent desensitization and internalization (Daigle et al., 2008a; Wickert et al., 2018; Al-Zoubi et al., 2019; Figure 5).

Several *in vitro* studies have examined the coupling of CB1R with β-arrestin 1 and/or 2, although results differ widely depending on cell type and agonist used (Jin et al., 1999; Daigle et al., 2008a, b; Gyombolai et al., 2013, 2015; Laprairie et al., 2014, 2016; Delgado-Peraza et al., 2016). Most recently, a study using bioluminescence resonance energy transfer (BRET) to measure β-arrestin translocation to the membrane following untagged CB1R activation in HEK293 cells showed stronger β-arrestin 2 membrane translocation than β-arrestin 1, although β-arrestin 2 coupling was, again, highly dependent on the specific ligand (Ibsen et al., 2019).

Functional *in vivo* studies using β-arrestin 1 or β-arrestin 2 knockout mice show that their roles in CB1R desensitization and tolerance are highly dependent on brain region and agonist type (Breivogel et al., 2008, 2013; Nguyen et al., 2012; Breivogel and Vaghela, 2015). Deletion of β-arrestin 1 modulates the effects of acute synthetic agonist CP55940, but not THC, on cannabinoid-mediated behaviors such as antinociception and hypothermia, but does not affect the development of tolerance to either CP55940 or THC (Breivogel and Vaghela, 2015). A similar agonist-dependent effect is seen in β-arrestin 2 knockout mice (Breivogel et al., 2008). Deletion of β-arrestin 2 enhances acute THC-mediated antinociception and hypothermia, but not catalepsy (Nguyen et al., 2012). Furthermore, with repeated administration of Δ^9^-THC, β-arrestin 2 knockout mice displayed reduced desensitization in cerebellum, caudal periaqueductal gray and spinal cord, along with attenuated tolerance to Δ^9^-THC-mediated antinocioception. However, β-arrestin 2 knockout mice showed *increased* desensitization in the hypothalamus, cortex, globus pallidus and substantia nigra, along with greater tolerance to THC-induced catalepsy (Nguyen et al., 2012). These data indicate that the role of β-arrestin 1 and 2 in CB1R desensitization and internalization is highly agonist and region specific (see Al-Zoubi et al., 2019 for a recent review).

β-arrestin binding sites usually include a cluster of at least 2 phosphorylated residues and they are typically recruited to GPCRs through GPCR kinase (GRK)-mediated phosphorylation (Moore et al., 2007). CB1R desensitization involves GPCR kinase 2/3 (GRK2/3)-mediated phosphorylation of two serine residues (S426 and S430), which recruit β-arrestin 1/2 and prevent G protein coupling (Jin et al., 1999; Kouznetsova et al., 2002; Daigle et al., 2008a; Morgan et al., 2014). Similar to deletion of β-arrestin 2, S426A/S430A knock in mice display reduced tolerance to cannabinoid-mediated pain for some agonists, but not others (Morgan et al., 2014; Nealon et al., 2019).

However, S426 and S430 phosphorylation are not sufficient for agonist-induced CB1R internalization, which requires phosphorylation of combinations of six serines and threonines at the extreme C-terminus that modulate β-arrestin 1/2 recruitment (Moore et al., 2007; Daigle et al., 2008b; Straiker et al., 2012b). In addition, pharmacological analysis has suggested that β-arrestin 1 and 2 may play distinct roles in CB1R signaling and internalization, respectively (Ahn et al., 2013a). Indeed, the cortex of β-arrestin 2 knockout mice contain more synaptosomal CB1R compared to WT mice (Breivogel et al., 2013) while β-arrestin 1 knockout mice show no difference in CB1R density in brain membranes compared to WT controls (Breivogel and Vaghela, 2015).

While a role for β-arrestins in CB1R function is well-established, the only studies to report a direct association of β-arrestins with CB1R used NMR spectroscopy to examine the interaction of purified β-arrestin 1 with phosphorylated ctCB1R peptides (Bakshi et al., 2007; Singh et al., 2011), and a complication that currently hampers definitive conclusions is that the effects of modifying S426 and S430, and other phosphorylation sites in ctCB1R, appear to be highly dependent on the cell type studied (Straiker et al., 2012b). Therefore, while it is clear that arrestins play an important role in the modulation of CB1R surface expression and signaling, the exact details of how and where on CB1R binding occurs remain to be determined.

#### G Protein-Coupled Receptor Associated Protein 1 (GASP1)

G protein-coupled receptor associated protein 1 has been linked to the intracellular sorting of CB1R to lysosomes following chronic exposure to agonist (Simonin et al., 2004). Members of the family of GASP proteins (GASP1-10) bind the C-terminal tails of multiple GPCRs, including the D4 dopaminergic receptor, the β2 adrenergic receptor, and the δ opioid receptor, where they play roles in post-endocytic sorting to lysosomes for degradation (Whistler et al., 2002; Heydorn et al., 2004; Simonin et al., 2004; Moser et al., 2010). A screen of a range of GPCRs has identified two conserved residues in the *H8* motif of CB1R (F409/R410) that are required for high-affinity GASP1 interactions (Simonin et al., 2004). Despite the fact that GASP1 was originally isolated as a CB1R interactor from a GST pulldown using only the extreme distal portion of ctCB1R [the last 14 amino acids; (Martini et al., 2007)], CB1R truncations lacking the last 13 amino acids of the C-terminal tail were still able to co-immunoprecipitate GASP1 when co-expressed in HEK293 cells (Tappe-Theodor et al., 2007), potentially via binding to *H8*. Thus, the precise binding domain for GASP1 on CB1R has not yet been identified and it is possible that there may be multiple sites for GASP1 interaction in ctCB1R.

Consistent with its known function, expression of a dominant negative GASP1 attenuated downregulation of surface CB1R induced by chronic (24 h) exposure to the agonist WIN55,212-2 in both HEK293 cells stably expressing N-terminally FLAG-tagged CB1R and in primary neurons (Martini et al., 2007; Tappe-Theodor et al., 2007). Importantly, this process has been implicated in the development of tolerance to cannabinoids in habitual cannabis users since, unlike wild-type mice, GASP1 knockout mice do not develop tolerance to repeated administration of WIN55,212-2 in 3 components of the classic tetrad of cannabinoid-mediated behaviors (antinociception, hypolocomotion, and catalepsy) (Martini et al., 2010). However, WT and GASP1 knockout mice both developed tolerance to WIN55,21202-mediated hypothermia, potentially due to differences in overlapping expression of CB1R and GASP1 in different brain regions driving diverse mechanisms of tolerance (Martini et al., 2010). However, one limitation of this study when extrapolating the results habitual cannabis users is the use of WIN55,212-2 rather than Δ^9^-THC since desensitization, internalization, recycling, and tolerance are highly agonist-specific (Hsieh et al., 1999; Wu et al., 2008; Martini et al., 2007; Morgan et al., 2014; Nealon et al., 2019). Indeed, a recent study reported that Δ^9^-THC- but not WIN55,212-2-mediated tolerance is dependent on c-Jun N-terminal Kinase (JNK) signaling (Henderson-Redmond et al., 2020).

#### Cannabinoid Receptor Interacting Protein 1a and 1b (CRIP1a/b)

Cannabinoid receptor interacting protein 1a (CRIP1a), and its primate-specific splice variant CRIP1b, represent a novel class of proteins that were identified by yeast-two hybrid screens against a human cDNA library using the last 55 amino acids of CB1R as a bait (Niehaus et al., 2007). Interaction with ctCB1R was validated *in vitro* using GST pulldowns of purified CRIP1a/b protein and co-immunoprecipitations from rat brain lysates (for CRIP1a only, as CRIP1b is primate specific), further suggesting that these proteins interact *in vivo* (Niehaus et al., 2007). Furthermore, in mice, CRIP1a co-localizes with CB1R at presynaptic boutons in both pyramidal neurons and interneurons of the hippocampus, and *in situ* hybridization analysis shows that CRIP1a and CB1R expression overlap, especially in glutamatergic and GABAergic neurons in the hippocampus (Guggenhuber et al., 2016). However, CRIP1a is also abundant in cells that express little or no CB1R (e.g., dentate granule cells), indicating that CRIP1a likely has other functions independent of CB1R (Guggenhuber et al., 2016).

Five amino acids in the very distal carboxy terminus of CB1R are necessary for CRIP1a binding: D467, T468, S469, A472, and L473 (Mascia et al., 2017) and, interestingly, a similar motif present in the metabotropic glutamate receptor mGlu8a also binds CRIP1a (Mascia et al., 2017).

Cannabinoid receptor interacting protein 1a (but not 1b) contains a Class I PDZ ligand at its C-terminus (Niehaus et al., 2007) indicating a possible interaction with PDZ domain-containing proteins. Furthermore, CRIP1a (but not 1b) contains a palmitoylation site that may facilitate partitioning to the membrane and thus association with CB1R (Niehaus et al., 2007; Booth et al., 2019).

It has been reported recently that CRIP1a overexpression suppresses agonist-induced internalization of CB1R, but not desensitization, by competing with β-arrestin-2 for binding to the distal tail of ctCB1R (Smith et al., 2015; Blume et al., 2017). Phosphorylation of ctCB1R at T468 reduced binding to CRIP1a, allowing for β-arrestin-2 binding (Blume et al., 2017). This is consistent with the requirement for phosphorylation of residues in the central region of ctCB1R for desensitization, but not internalization, which requires phosphorylation of the distal tail (Jin et al., 1999; Kouznetsova et al., 2002; Daigle et al., 2008b; Straiker et al., 2012a; Morgan et al., 2014). Accordingly, overexpression of CRIP1a attenuates CB1R G protein signaling in HEK293 cells, N18TG2 cells, and autaptic neuronal cultures, reducing the downstream inhibition of N-type VGCCs and activation of ERK (Niehaus et al., 2007; Blume et al., 2015, 2017; Smith et al., 2015).

#### Src Homology 3-Domain Growth Factor Receptor-Bound 2-Like (endophilin) Interacting Protein 1 (SGIP1)

SGIP1 was first identified as a novel transcript in a screen of hypothalamic mRNA in the obesity model of the fat sand rat (*Psammomys obesus*) that is markedly upregulated in comparison to lean counterparts (Trevaskis et al., 2005). Both SGIP1 and CB1R are strongly associated with diet-induced obesity [DIO; (Trevaskis et al., 2005; Soria-Gomez et al., 2014; Cardinal et al., 2015)] and siRNA-mediated knockdown of hypothalamic SGIP1 inhibited food intake, suggesting that SGIP1 in the hypothalamus plays a role in energy expenditure (Trevaskis et al., 2005).

Since its identification, accumulating evidence has identified SGIP1 as an endocytic protein. SGIP1 is capable of binding liposomes (Uezu et al., 2007), and also interacts with a number of adaptor proteins with roles in CME, including endophilin 3 (Trevaskis et al., 2005) the AP-2 complex (Hollopeter et al., 2014) intersectin 1 (ITSN1) (Dergai et al., 2010) amphiphysin, (Daumke et al., 2014) and Eps15 (Uezu et al., 2007).

SGIP1 was identified as a CB1R interactor by yeast two-hybrid assays using ctCB1R as a bait, and the interaction confirmed using coimmunoprecipitation assays (Hajkova et al., 2016). Moreover, SGIP1α, a brain-specific isoform of SGIP1, is enriched in presynaptic boutons (Wilhelm et al., 2014), and co-localizes with CB1R, bassoon, and synaptotagmin 1 (Hajkova et al., 2016; Lee et al., 2019).

Importantly, co-expression of SGIP1 with CB1R in HEK293 cells interfered with agonist-induced internalization of CB1R compared to cells expressing CB1R alone (Hajkova et al., 2016). This reduction in agonist-induced internalization was almost the same as the reduction in internalization that occurred when CME was blocked by expression of a dominant negative dynamin. A smaller, but significant, reduction in constitutive internalization was also observed in CB1R/SGIP1 co-expressing cells compared to HEK293 cells expressing CB1R alone. Furthermore, SGIP1 enhanced β-arrestin-2 association with activated CB1R and reduced CB1R agonist-induced ERK1/2 activation. However, G_i/o_-protein activation and downstream Ca^2+^ mobilization were unaffected (Hajkova et al., 2016).

Since SGIP1 is part of the CME complex it is initially counter-intuitive that its overexpression *prevents* CB1R internalization. An explanation for this is that SGIP1 competes for binding with FCHo1/2 proteins (Hajkova et al., 2016). Both SGIP1 and FCHo1/2 are members of the muniscin family of cargo adapters, which contain an N-terminal membrane biding domain, an AP-2 activator domain, a proline rich domain, and a C-terminal μ homology domain (Dergai et al., 2010; Hollopeter et al., 2014). However, the membrane binding domain of FCHo1/2 is an F-BAR domain capable of plasma membrane shaping during pit formation, while the membrane binding domain of SGIP1 has no sequence similarity. Thus, differential membrane binding may allow SGIP1 to act as “dominant negative” to inhibit FCHo1/2-dependent CB1R internalization. Furthermore, SGIP1 interacts with endophilin 3, which has also been reported to inhibit endocytosis (Sugiura et al., 2004; Zhang et al., 2015). It should be noted, however, that SGIP1 knockdown has been reported to selectively *impair* internalization of the pre-synaptic protein synaptotagmin 1 during synaptic vesicle recycling, although other synaptic vesicle proteins including synaptophysin and VAMP2 were not affected by SGIP1 knockdown (Lee et al., 2019). SGIP1 also reportedly activates AP-2 (Hollopeter et al., 2014) and initiates membrane tubulation, and both SGIP1 overexpression and knockdown reduce transferrin (Tfn) uptake (Uezu et al., 2007).

Thus, SGIP1α is an endocytic adaptor protein that appears to play complex, and potentially target specific, roles in plasma membrane protein internalization. Nonetheless, it selectively reduces CB1R internalization and since both CB1R (Mazier et al., 2015) and SGIP1α (Trevaskis et al., 2005; Cummings et al., 2012) play key roles in food intake and energy expenditure (Hajkova et al., 2016), it is interesting to speculate that targeting the interaction between these two proteins could be a useful therapeutic approach to combat obesity.

## Overview and Perspectives

There is burgeoning medical and societal interest in the recreational and therapeutic use of cannabinoid drugs. In addition, there is intense scientific interest in, and appreciation of, the pervasive influence and importance of ECS neuromodulatory feedback in almost all aspects of synaptic transmission and plasticity. However, although the complex pharmacology has been the subject of concerted research for decades, our knowledge of the protein interactions and synaptic dynamics of cannabinoid receptors lags far behind that of many other neurotransmitter receptors, and there remain many outstanding questions.

Indeed, the question of whether CB1R is directly axonally targeted from the secretory pathway has produced confounding results, with some studies favoring a direct route, and others observing non-targeted CB1R sorting at the TGN. While the differences between these studies may result from different methodological approaches, ages of neurons used and differently tagged receptors, further work will be required to resolve these discrepancies. Similarly, while there is a broad agreement that differential endocytosis rates between axons and the somatodendritic compartment contributes to the axonally polarized surface expression of CB1R, there is conflicting evidence regarding the role of receptor conformation and basal activity in these processes. Moreover, while the protein interactions and post-translational modifications that control CB1R signaling, internalization and sorting have begun to be addressed, these studies have utilized a number of different experimental systems that have made full interpretation of these findings difficult. Undoubtedly, further studies on these issues, using standardized approaches where possible and focusing on these pathways in neuronal cells, will benefit and add clarity to the field. Finally, while a number of CB1R interactors have been identified, there almost certainly exist a variety of others than are currently unknown. Future studies using both targeted and mass spectrometry approaches will likely address these exciting possibilities directly.

Given the importance of the ECS in a wide variety of brain functions, and its implication in a number of disease states, we anticipate that this fundamental “nuts and bolts” knowledge will both provide important information about the function and organization of the ECS, and potentially uncover novel therapeutic targets for beneficially manipulating CB1R function in a number of contexts.

## Author Contributions

All authors listed have made a substantial, direct and intellectual contribution to the work, and approved it for publication.

## Conflict of Interest

The authors declare that the research was conducted in the absence of any commercial or financial relationships that could be construed as a potential conflict of interest.
