# Corrigendum: Protein Interactors and Trafficking Pathways That Regulate the Cannabinoid Type 1 Receptor (CB1R)

**DOI:** 10.3389/fnmol.2020.00142

**Published:** 2020-08-21

**Authors:** Alexandra Fletcher-Jones, Keri L. Hildick, Ashley J. Evans, Yasuko Nakamura, Jeremy M. Henley, Kevin A. Wilkinson

**Affiliations:** Centre for Synaptic Plasticity, School of Biochemistry, University of Bristol, Bristol, United Kingdom

**Keywords:** endocannabinoid system, cannabinoid type 1 receptor, trafficking, protein-protein interactions, synaptic regulation, retrograde synaptic signaling

In the original article, we neglected to include the funders **BBSRC, (BB/R00787X/1)** to **JH and KW** and **Wellcome Trust, (105384/Z/14/A) to JH and AE**.

The authors apologize for this error and state that this does not change the scientific conclusions of the article in any way. The original article has been updated.

